# Behavioral Dynamics, Genomic Insights, and Social Drivers of SARS-CoV-2 Waves and Variants in Cali, Colombia (2020–2023)

**DOI:** 10.3390/v17060800

**Published:** 2025-05-30

**Authors:** Diana López-Alvarez, Nelson Rivera-Franco, Erica Aristizabal, Melissa Solarte, Andrés Castillo, Carlos A. Pardo, Beatriz Parra

**Affiliations:** 1 Grupo VIREM—Virus Emergentes y Enfermedad, Departamento de Microbiología, Escuela de Ciencias Básicas, Facultad de Salud, Universidad del Valle, Cali 760043, Colombia; rivera.nelson@correounivalle.edu.co (N.R.-F.); erica.aristizabal@correounivalle.edu.co (E.A.); melissa.solarte.cadavid@correounivalle.edu.co (M.S.); beatriz.parra@correounivalle.edu.co (B.P.); 2 Departamento de Ciencias Biológicas, Facultad de Ciencias Agropecuarias, Universidad Nacional de Colombia, Palmira 763533, Colombia; 3 Laboratorio de Técnicas y Análisis Ómicos—TAOLab/CiBioFi, Facultad de Ciencias Naturales y Exactas, Universidad del Valle, Calle 13 No 100-00, Cali 760015, Colombia; andres.castillo.g@correounivalle.edu.co; 4 Department of Neurology, Johns Hopkins School of Medicine, Baltimore, MD 21287, USA; cpardov1@jhmi.edu; 5 Department of Pathology, Johns Hopkins School of Medicine, Baltimore, MD 21287, USA

**Keywords:** genomic surveillance, mutations, epidemic waves, social transmission

## Abstract

In Cali, Colombia, 405,689 COVID-19 cases were reported until March 2023, with 2463 complete genome sequences available for analysis. SARS-CoV-2 genomic data from Cali were analyzed to determine the prevalence of variants as well as the mutation frequencies. This study identified Nextstrain clades, Pango lineages, and specific mutations in key viral proteins. A total of 23 Nextstrain clades and 118 Pango lineages were detected, including variants of interest (Lambda, Mu) and variants of concern (Alpha, Gamma, Delta, Omicron). Analysis identified 2424 missense mutations, with notable frequencies in NSP3 (465), S (367), NSP2 (205), N (180), ORF3a (144), NSP12b (113), and NSP13 (108). The study also observed a high prevalence of simultaneous transmission of multiple variants. The COVID-19 epidemic waves in Cali were shaped more by social and economic dynamics than by the emergence of specific SARS-CoV-2 variants. These findings highlight the importance of context-specific public health interventions to mitigate future outbreaks effectively.

## 1. Introduction

Severe acute respiratory syndrome coronavirus 2 (SARS-CoV-2), responsible for the COVID-19 pandemic, was first recorded in Wuhan, China, in late 2019 and put global healthcare systems to the test, including those in Latin America. This underscored the urgent need for genomic surveillance to detect imported cases and limit transmission [1]. Colombia, particularly affected among South American countries, experienced a significant surge in COVID-19 cases, with the virus’s evolution and epidemic spread widely studied within the country [2,3]. Portable sequencing technologies, such as Oxford Nanopore [4,5], were rapidly and effectively implemented to monitor viral evolution. Among South American countries, Colombia was among the most severely impacted [6], ranking 22nd among 187 countries in COVID-19 deaths per 100,000 people as of February 2022 [7]. By March 2023, Colombia had reported over 5.8 million COVID-19 cases and 142.722 associated deaths [8]. The State of Valle del Cauca was one of the most affected regions, ranking third in case numbers, with 569,458 reported cases and 15,458 deaths. Cali, the state capital, reported 405,689 cases and 8926 deaths during the same period, representing 71.2% of the state’s total cases [8].

During that period, mutated SARS-CoV-2 lineages emerged worldwide due to the rapid spread of the virus and evolution [9]. Numerous lineages and variants have been described since the beginning of the COVID-19 pandemic [10]. The first ‘variants of concern’ (VOCs) [11] identified was the Alpha variant (B.1.1.7 lineage), detected in the United Kingdom in September 2020 [12]. This was followed by Beta (B.1.351) and Gamma (P.1), both identified in November 2020 in Brazil; Delta (B.1.617.2 + AY.x), first detected in India in December 2020; and Omicron (BA.1 BA.2). These variants exhibited mutations in the spike protein, potentially increasing transmissibility and immune evasion [13]. Among ‘variants of interest’ (VOIs), several emerged in South America, including the Mu variant (B.1.621 lineage) and Gamma variant (P.1), which were detected in January 2021 in Colombia [14,15]. Lambda variant (C.37 lineage) was first reported in Peru in August 2020 [16], and in Colombia on 6 March 2020 [17]. Conversely, the Alpha variant (B.1.1.7) was detected in February 2021 in the state of Caldas [18], and the Delta variant (B.1.617.2) in July 2021, detected in a specifically identified traveler from the United States (US) in Cali [19]. The Omicron variant was first identified in December 2021 in the cities of Santa Marta and Cartagena, detected in three travelers—two from the US and one from Spain [20]. Additionally, the Lambda variant (C.37 lineage) was detected in May 2021 in the state of Boyacá [21]. Currently, variants such as Alpha (B.1.1.7 and Q lineages), Beta (B.1.351 and descendant lineages), Gamma (P.1 and descendant lineages), Delta (B.1.617.2 and descendant lineages), Mu (B.1.621, B.1.621.1), and several Omicron lineages have changed classification and are now categorized as ‘variants being monitored’ (VBMs) [11].

We monitored the local spread of SARS-CoV-2 in Cali, Colombia, over three years of the pandemic (March 2020–March 2023) using 2463 genome sequences. As part of our effort to support global SARS-CoV-2 variant surveillance, we contributed to the early reporting of the first SARS-CoV-2 genome from Cali, Colombia (EPI_ISL_445219).

## 2. Materials and Methods

### 2.1. Sample Collection, Library Construction, and Sequencing Methods

Nasopharyngeal swabs or aspirate specimens were obtained from patients admitted to various healthcare facilities in Cali and placed in viral transport medium (VTM) following WHO protocols. Automated RNA extraction was performed on 200 µL of VTM using the MagMAX™ Viral/Pathogen Nucleic Acid Isolation Kit for KingFisher Flex from Thermo Fisher Scientific Inc. (Waltham, MA, USA), following the manufacturer’s guidelines. All samples underwent RT-qPCR analyses to assess the relative abundance of viral RNA, targeting two regions of the Nucleocapsid (N1 and N2), with the human RNase P gene (RP) serving as a control, as outlined in the CDC 2019-Novel Coronavirus (2019-nCoV) Real-Time RT-PCR Diagnostic Panel (2020) [22]. Samples with N2 Ct values between 5 and 25 were selected for sequencing, and the extracted RNA was subsequently preserved at −80 °C until sequencing library preparation.

Sequence libraries were prepared following the ARTIC Network protocol [23]. cDNA was synthesized from viral RNA using the LunaScript^®^ RT SuperMix Kit (Cat #: E3010G) from New England Biolabs-NEB (Ipswich, MA, USA). The cDNA was then amplified using Q5 high-fidelity DNA polymerase (NEB; Cat #: M0492) and ARTIC v3 primers from Integrated DNA Technologies, Inc.-IDT (San Diego, CA, USA). For Omicron genomes, ARTIC v4.1 primers (IDT) were used instead. The resulting 400 bp amplicons were purified using Agencourt AMPure XP beads (A63881) from Beckman Coulter, Inc. (Brea, CA, USA) (and quantified with a Qubit fluorometer using a Qubit™ dsDNA HS Assay kit (Thermo Fisher Scientific, Inc.). Library preparation was performed using a SQK-LSK109 ligation sequencing kit, and sequencing was carried out on a MinION MK1B instrument from Oxford Nanopore Technologies PLC (Oxford, UK) with FLO-MIN106D (R9.4.1) flow cells (Oxford Nanopore Technologies).

### 2.2. Viral Genome Assembly and Genome Sequence Dataset Retrieval

This study analyzed 2463 complete SARS-CoV-2 genomes from Cali, Colombia, obtained from the Global Initiative on Sharing All Influenza Data (GISAID, https://www.gisaid.org; accessed on 21 April 2023), of which 1414 SARS-CoV-2 genomes (57.4%) were produced in-house by this study as described above and deposited on GISAID in collaboration with the National Genomic Surveillance of SARS-CoV-2 Program of the Colombian National Institute of Health (INS). Nanopore sequencing data (Fast5) were base-called and demultiplexed using Guppy v4.2.2, except for samples from 2022, which were processed exclusively with Guppy v6.2.1. The genome assembly was performed using the ARTIC Network bioinformatics protocol v.1.2.1 [23] with the recommended settings. Quality control and read filtering (400 to 700 base pairs) were performed using the Artic Guppyplex filter script. Consensus variants were identified using Medaka, with the Wuhan-Hu-1 isolate (GenBank accession number MN908947.3) as the reference. Each assembled genome was classified according to the Pangolin nomenclature for SARS-CoV-2 lineages and Nexclades for SARS-CoV-2 clades (https://clades.nextstrain.org/; accessed on 17 November 2024).

### 2.3. Number of Cases, Infection Fatality Rate (IFR), Vaccination, and Effective Reproduction Number (Re)

We compared the frequency of detected clades with the number of positive cases and deaths reported from Cali during the same period. These epidemiological data were obtained from the INS database (accessed on 22 December 2024). The effective reproduction number (Re) was estimated using the EpiEstim package v.2.2.4 in R [24]. This package employs an incidence curve-based approach to estimate Re, representing the average number of new cases generated by an infected individual. The estimation assumed a parametric distribution for the serial interval (SI) (the time between a person’s infection and the infection of their secondary contacts), using parameters from the ‘COVID-19 Estimator’ developed by the Pan-American Health Organization (PAHO) and World Health Organization (WHO) (https://harvardanalytics.shinyapps.io/covid19/): a mean serial interval of μ_si_ = 4.8 days and a standard deviation serial interval of σ_si_ = 2.3 days. Re was calculated over time, using weekly moving windows, and presented with 95% confidence intervals.

The Infection Fatality Rate (IFR) was calculated by dividing the number of deaths by the number of confirmed positive cases each month. Data on daily COVID-19 vaccinations and the distribution of vaccination statuses were obtained from publicly available records of the Mayor’s Office of Cali [25,26].

### 2.4. Phylogenetic Analysis and Mutation Site Identification

A total of 2463 sequences were analyzed from March 12, 2020, to March 7, 2023. Phylogenetic analysis was conducted using the Nextstrain pipeline (https://github.com/nextstrain/ncov; accessed on 22 November 2024) [27]. The genomes obtained from Cali were aligned by MAFFT [28] alongside the Wuhan-Hu-1 reference genome. Maximum likelihood phylogenetic inference was performed with IQ-TREE [29], and molecular clock calibration was conducted using TreeTime [30]. The results, in JSON format, were interactively explored on the auspice.us web platform. Alignment files were further analyzed using the online COVID-19 genome annotator program (http://giorgilab.unibo.it/coronannotator/, accessed on 30 November 2024). Single-nucleotide variant (SNV) frequencies and non-synonymous mutation counts were extracted using R.

## 3. Results

### 3.1. Demographic Distribution

A total of 2463 whole-genome sequences of SARS-CoV-2 from Cali, Colombia, were analyzed to characterize genomic variants. Most samples were from female patients (54.9%), and working-age individuals (18–60 years) comprised 66.9% of the total samples (Table S1).

**Figure 1:**
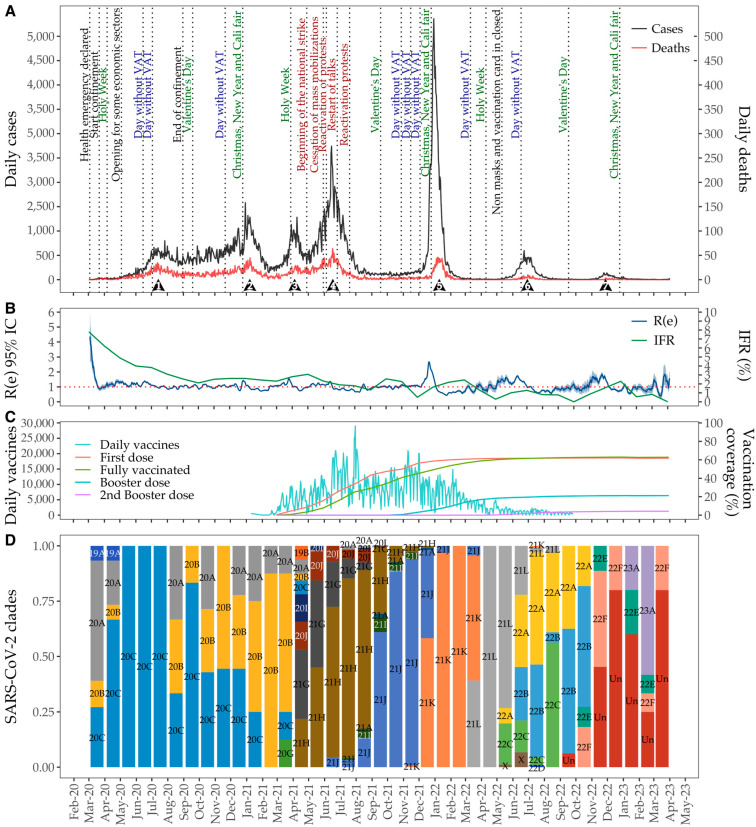
Dynamics of SARS-CoV-2 from 1 March 2020 to 7 March 2023 in Cali. (**A**) Number of confirmed cases (black line) and deaths (red line). Dotted vertical lines indicate key social, economic, public order, and pandemic management events. (**B**) Infection Fatality Rate (IFR) (green line) and estimate of the effective reproductive number (Re) (blue line). (**C**) Daily administered vaccination doses (cyan line) and cumulative vaccination coverage: first dose (red line), second dose (blue line), first booster (green line), and second booster (purple line). (**D**) Time series of SARS-CoV-2 genome identification by clade.

Cali experienced seven distinct ‘waves’ during the COVID-19 epidemic: June–August 2020 (34.334 cases and 1.289 deaths), November 2020–February 2021 (77,164 cases and 1956 deaths), March–April 2021 (28.818 cases and 769 deaths), May–August 2021 (107,415 cases and 2559 deaths), December 2021–February 2022 (93,259 cases and 1064 deaths), May–July 2022 (15,164 cases and 159 deaths), and November 2022–January 2023 (3859 cases and 52 deaths). These waves varied in duration, peak intensity, and mortality over time (Figure 1A). The peak reproductive number (Re) reached its highest value on zero day, following by the fifth wave before rapidly declining (Figure 1B).

The initial surge in infections was closely preceded by the implementation of economic revitalization measures in the city between May 2020 and June 2020. These measures included the reopening of specific economic sectors and two full tax-free shopping days. Subsequent waves, notably the second and fifth, coincided with the Christmas holidays in December, the Cali city-fair, and the New Year celebrations. The third wave followed the Holy Week, while the fourth corresponded with a period of widespread social protests. The final, smaller waves (sixth and seventh) emerged after the discontinuation of mask mandates and the introduction of vaccination card requirements for enclosed spaces (Figure 1A).

The IFR was highest at the onset of the pandemic in May 2020. It then gradually declined until October 2020, followed by fluctuations through December 2021. Notably, December recorded one of the lowest mortality rates, comparable to those observed in May 2022 (Figure 1B). As shown in Figure 1B, the reproductive number (Re) peaked at the beginning of the pandemic in Cali (Re: 4.37, SD: 0.82), followed by the end of the month. Throughout the rest of the study period, Re values remained close to 1, with minor increases in January 2021 (Re: 1.44, SD: 0.017), April 2021 (Re: 1.56, SD: 0.021), and July 2021 (Re: 1.36, SD: 0.011). More pronounced spikes were observed in January 2022 (Re: 2.68, SD: 0.029), June 2022 (Re: 1.73, SD: 0.050), November 2022 (Re: 1.84, SD 0.095), and March 2023 (Re: 1.85, SD: 0.356).

In February 2021, Cali launched its COVID-19 vaccination program, administering a median of 4572 doses per day (ranging from 1 to 29,028), with a total of 3,511,434 doses administered. By September 2021, 50% of the population had received at least one dose, and many had completed full vaccination by December 2021. By July 2022, approximately 83% of the population had received at least one dose, while 75% had completed the full vaccination schedule. Booster doses were introduced in December 2021 (first booster) and April 2022 (second booster). As of July 2022, about 31% of the population had received the first booster, 3% had received a first booster, and 3% had received a second booster (Figure 1D).

Using available nomenclature systems, we identified 118 distinct Pango lineages (Figure 1 and Table S2) and 23 clades using Nextclade (Figure 1 and Table S3). During the early phase of the epidemic (March 2020–March 2021), ancestral clade lineages were observed as follows: 19A (*n* = 2; 0.1%), followed by 20A (Lineages B.1, B.1.111, B.1.36.19, B.1.420, and B.1.625) (*n* = 25; 1%), 20B (Lineages B.1.1 and B.1.1.348) (*n* = 32; 1.3%), 20C (Lineages B.1, B.1.623 and B.1.637) (*n* = 58; 2.4%), 20G (Lineages B.1.2) (*n* = 1; 0.04%), and 21J (Lineages AY.101) (*n* = 1; 0.04) (refer to Table S1 for details).

As of April 2021, clade 20I (Alpha.V1, B.1.1.7) was observed (*n* = 9; 0.4%). This was followed by clade 20J (Gamma.V3), which included lineages P.1., P.1.15, and P.1.7) (*n* = 35; 1.4%), 21G (Lambda, C.37) (*n* = 65; 2.6%), and 21H (Mu, including lineages B.1.621, B.1.621.1, and B.1.621.2) (*n* = 438; 17.8%), all of which circulated until September 2021. The Delta variant subsequently emerged with clades 21J (comprising 21 lineages, see Table S2) (*n* = 473; 19.2%), first detected in Cali. This was followed by clade 21I (Lineage AY.24., AY.26, AY.47, and AY.9.2) (*n* = 17; 0.7%) and clade 21A (Lineage AY.14., AY.35, AY.49, and B.1.617.2) (*n* = 2; 0.1%), all of which exhibited high circulation between October and December 2021. In November 2021, clade 21K (including nine lineages by Omicron BA.1, *n* = 470; 19.1%) emerged, progressively replacing the Delta variant until July 2022. Other notable clades circulating during this period and until March 2023 included Clade 21L (9 lineages by Omicron BA.2, *n* = 105; 4.3%), clade 22A (5 lineages by Omicron BA.4, *n* = 143; 5.8%), clade 22B (26 lineages by Omicron BA.5, BE, and BF, *n* = 138; 5.6%), clade 22C (Lineages BA.2.12.1 and BG.2, *n* = 37; 1.5%), clade 22D (Lineage BA.2.75.3, *n* = 1; 0.04%), clade 22E (Lineages BQ.1, BQ.1.1, BQ.1.1.4, BQ.1.1.10, and BQ 1.3, *n* = 18; 0.7%), clade 22F (Lineages XBB.1.15 and XBB.1.15.1, *n* = 52; 2.1%), and clade 23A (Lineages XBB.1.5, XBB.1.5.76, and XBB.1.5.77, *n* = 22; 0.9%). Finally, two recombinant variants, XAM (0.2%) and XAP (0.1%), were identified.

Among the months exhibiting the highest diversity, April 2021 stood out with a representation of eight Pango lineages and eight clades. August 2021 also demonstrates notable diversity, featuring 23 Pango lineages and eight clades. However, concerning clades, September 2021 was the most diverse month with 28 distinct lineages. It is noteworthy that from October 2021 to December 2021, an average of 31 Pango lineages were identified. This period was marked by the widespread circulation of diverse lineages predominantly associated with the Delta variant (refer to Tables S2 and S3). Finally, from January 2022 until March 2023, 74 Pango lineages were detected, all related to the Omicron variant.

The dominance of SARS-CoV-2 lineages evolved over time. Initially, between March and July 2020, lineage B.1 was highly prevalent, accounting for 83.63% of the cases. This was followed by new variants, such as B.1.1.348 (41.6%) and B.1.111 (12.5%), becoming predominant from August 2020 to February 2021. By April 2021, a significant shift occurred with the rise of lineages C.37 (31.3%), P.1 (12.5%), and B.1.621 (21.9%). Among these, B.1.621 reached its peak prevalence in July 2021, comprising 80.3% of the cases. Although it remained dominant until August 2021, it was eventually replaced by multiple Delta lineages in September 2021. Notably, AY100 (12.6%) and AY.25.1 (10.6%) emerged as leading variants. Overall, September 2021 saw the identification of 24 distinct Delta lineages, which collectively accounted for 69.3% of cases, marking a significant transition in the viral landscape.

By October and November 2021, the predominant SARS-CoV-2 variants in Cali were AY.20, AY.43, and AY.25.1. However, this dominance shifted with the emergence of B.A.1.1 (Omicron), which became the most prevalent variant from December 2021 to March 2022 (Table S1). In March 2022, B.A.2 (Omicron) appeared and remained dominant through April and May 2022. Subsequently, from June to October 2022, BA.4.1 (Omicron) and BA.5.6 became the most prevalent variants, with frequencies of approximately 33.2% and 17.8%, respectively. Finally, from November 2022 to March 2023, the dominant circulating variants were XBB.1.15 (55.6%), XBB.1.5 (12.2%), and XBB.1.5.77 (11.1%), marking the latest phase of variant evolution in the region.

### 3.2. Phylogenetic Analysis

**Figure 2:**
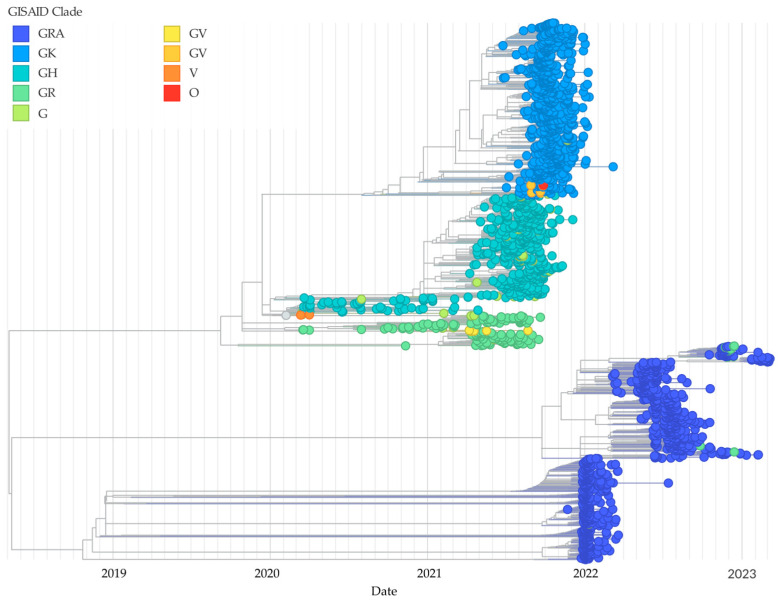
The phylogenetic tree analysis of 2463 genomic sequences in Cali, Colombia, categorized by clade distribution and collection date. Different clades (G, GH, GK, GR, GRA, GRY, GV, V, and O) are represented using different color codes. The phylogenetic tree was generated using the Nextstrain platform and Augur.

The phylogenetic analysis, consistent with Nextstrain (Figure 2), classified SARS-CoV-2 sequences into multiple clades, including G, GH, GK, GR, GRA, GRY, GV, V, and O. Among these, the most dominant clade was GRA (39.3%), followed by GK (31.8%) and GH (21.1%). Clades GR (5.8%) and G (1.5%) had a lower prevalence.

**Figure 3:**
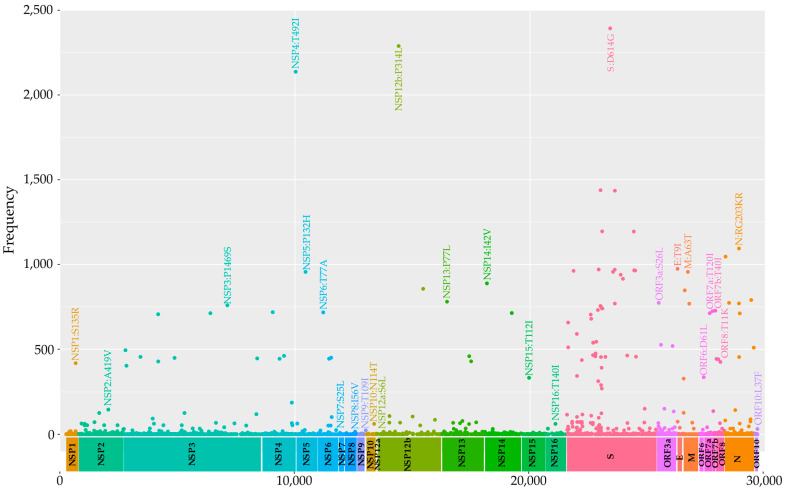
The distribution of the topmost frequent missense mutations in the virus genome. Dots indicate the genomic position of the missense mutations in each gene, which are represented in different colors.

Compared to the Wuhan reference genome, the genomes exhibited 4022 mutations, of which 2424 were missense mutations distributed across various genes (Figure 3). The seven most frequently mutated genes were NSP3 (465 mutations), S (367 mutations), NSP2 (205 mutations), N (180 mutations), ORF3a (144 mutations), NSP12b (113 mutations), and NSP13 (108 mutations). Remarkably, five spike protein mutations were detected, present in approximately 97,16% to 48,48% of the samples: D614G, T478K, P681H, N501Y, and D950N. Fifteen additional spike mutations were observed at intermediate frequency (~36%), namely, D1146D, L452R, N679K, Q954H, N969K, T95I, H655Y, N764K, A67, D796Y, P681R, S477N, E484A, Q498R, and Y505H. Finally, 28 mutations were found at low frequency (~19.6%), namely, N440K, G339D, R346K, T19R, E156, G142D, K417N, S373P, S375F, L24, T19I, R408S, I68, S371F, T376A, N856K, D405N, L981F, T547K, E484K, V213G, Q493R, T144, Y145N, G446S, H69, F486V, and G496S were observed.

## 4. Discussion

The dynamic progression of the COVID-19 pandemic in Cali was marked by seven distinct epidemic waves. Each wave was preceded by distinct social and economic events, which may have impacted the effective reproductive number (Re) and shaped the subsequent waves. Our study revealed a marked coincidence in timing between the COVID-19 waves and major societal events that promoted crowding and close social interactions, such as economic reopening measures, holidays, and social protests. This highlights the crucial role of human behavior and policy decisions in shaping the course of the pandemic, underscoring the need for context-specific public health interventions.

Humans play a critical role in the spread of infectious diseases. Fear of illness often encourages adherence to protective measures, but it can also coexist with optimism bias, causing underestimated risks and prematurely relaxed precautions, ultimately undermining public health efforts [31]. In Colombia, a combination of societal and systemic factors contributed to sustained COVID-19 surges. The gradual easing of preventive measures aimed at stimulating the economy coincided with pandemic fatigue and a strong public desire to return to normalcy. As a result, risk perception shifted—social issues such as hunger, unemployment, and violence became more pressing concerns than the threat posed by SARS-CoV-2 [2,31]. This divergence in perception and behavior leads to varied responses to health risks. While some individuals adhere strictly to enhanced social distancing measures, others might return to pre-pandemic social behaviors sooner than recommended. Premature relaxation of protective measures can create favorable conditions for disease transmission, potentially weakening the effectiveness of public health interventions [32]. Genomic surveillance of SARS-CoV-2 has been critical for tracking the real-time spread of emerging variants, providing invaluable insights into the dynamics of the COVID-19 pandemic. In Colombia, a total of 188 SARS-CoV-2 Pango lineages were documented between March 2020 and March 2023. In particular, 118 of these lineages were identified in Cali, representing 62.8% of the city’s reported cases. This comprehensive genomic tracking underscores the importance of continuous monitoring to assess and mitigate the effects of behavioral changes on disease transmission.

The first wave of COVID-19 cases occurred between June and August 2020, coinciding with the circulation of Pango lineage B.1, associated with clade 20C. This surge followed the reopening of economic sectors and two tax-free shopping days, which led to crowding in supermarkets and stores, increasing viral transmission. The second (November 2020 to February 2021) saw a rise in Pango lineage diversity. This increase in cases followed the end of the lockdown and peaked around the year-end holidays. Despite this diversity, lineage B.1 remained dominant, followed by B.1.1.348, which carries key spike mutations (D614G, R346K, S373P, G1167A). Notably, R346K, a spike protein mutation, is particularly significant as it is linked to increased transmissibility [33]. The third wave (March and April 2021) involved 12 distinct Pango lineages and coincided with Holy Week, a period of religious celebration and travel. The C.37 (Lambda) variant (clade 21G) was predominant, featuring L452Q and F490S mutations in the receptor binding region. F490S has been associated with reduced antibody neutralization [34]. The fourth wave of COVID-19 cases (May and August 2021) was primarily driven by the Mu variant (B.1.621 lineage, 21H clade). This period coincided with widespread social protests, leading to the highest number of positive cases recorded. The B.1.621 lineage is characterized by mutations as T95I, Y144T, Y145S, and a 146N insertion in the NTD, as well as R346K, E484K, and N501Y in the RBD and P681H at the S protein cleavage site [35]. While early stages suggest potential vaccine resistance, further research is needed to fully elucidate its impact on vaccine efficacy [36]. The fifth wave (December 2021–February 2022) was dominated by the Omicron variant, specifically the Pango BA.1.1 (clade 21K). A defining feature of BA.1.1 is the R346K mutation in the spike protein, which distinguishes it from BA.1, as the latter lacks this mutation [37]. This surge coincided with Christmas and New Year’s celebrations and the Cali Fair, which facilitated mass gatherings and rapid viral transmission, leading to unprecedented case numbers. The sixth and seventh waves peaked between May and July 2022 and November 2022 to January 2023, respectively. The last two waves were driven by Omicron subvariants, with BA.4.1 (clade 22A) responsible for the sixth wave, while XBB.1.15 (clade 22F) drove the seventh. The mask mandate was lifted before the sixth wave, but with 80% of the population vaccinated, these waves were less severe regarding case numbers.

Unlike previous studies that focused on short-term trends or variant-driven transmission dynamics [38,39,40], our research covers a longer timeframe and integrates epidemiological, social, and genomic data. This comprehensive approach reveals a consistent coincidence between periods of heightened social interaction—such as holidays, economic reopening, and social protests—and subsequent COVID-19 surges. These findings underscore the importance of accounting for behavioral and social context in pandemic preparedness and response strategies.

Strengths and limitations: This study stands out for its longitudinal scope; high-resolution genomic dataset (*n* = 2463); and integration of epidemiological metrics such as Re, IFR, and vaccination coverage across three years of the pandemic in a major urban center of Colombia. However, limitations include a potential sampling bias toward hospitalized or symptomatic cases, underrepresentation of rural areas, and a lack of fine-grained metadata (e.g., clinical outcomes or comorbidities), which may restrict the generalizability of findings and the ability to assess variant-specific virulence or vaccine effectiveness.

## 5. Conclusions

This study shows that increased social interaction frequently anticipated subsequent COVID-19 waves. This pattern was evident across diverse events, including holidays, religious gatherings, economic reopening, and social protests. Spanning March 2020 to March 2023, this study also documented the emergence of diverse SARS-CoV-2 variants, highlighting the dynamic nature of the pandemic. Importantly, Cali’s extensive genomic surveillance, accounting for 62.8% of Colombia’s Pango lineages, underscores the importance of continuous monitoring. While later waves coincided with relaxed mask mandates, the high vaccination rate (~ 80%) likely mitigated the outbreak severity. These findings emphasize the need for public health messaging that promotes responsible behavior during periods of increased social activity while also recognizing the critical role of vaccination in controlling future waves.

## Data Availability

The data used for this work can be accessed in the GISAID Initiative.

## Supplementary Materials

The following supporting information can be downloaded at https://www.mdpi.com/article/10.3390/v17060800/s1. Table S1. Patient demographics and specimen information for SARS-CoV-2 samples. Patient demographics, specimen details, and virus classification for SARS-CoV-2 samples obtained from patients in Colombia. It includes patient age, status, specimen type, host information, and Nextclade classification. Table S2. Monthly SARS-CoV-2 Pango lineage distribution. Monthly SARS-CoV-2 lineage distribution. Monthly distribution of SARS-CoV-2 Pango lineages from March 2020 onward, detailing the relative frequency of each variant detected over time along with total counts per month. Table S3. Monthly SARS-CoV-2 Nextclade lineage distribution. Monthly distribution of different SARS-CoV-2 Nextclade lineages over time, from March 2020 to March 2023. Table S4. Amino acid substitutions in SARS-CoV-2 proteins. Amino acid substitutions found in various SARS-CoV-2 proteins, along with the frequency of each substitution.

## Abbreviations

The following abbreviations are used in this manuscript:

SARS-CoV 2	Severe acute respiratory syndrome coronavirus 2
COVID-19	Coronavirus disease 2019
VOCs	Variants of concern
VOIs	Variants of interest
VBMs	Variants being monitored
VTM	Viral transport medium
RP	RNase P gene
N	Nucleocapsid
Ct	Cycle threshold
WHO	World Health Organization
INS	Colombian National Institute of Health
IFR	Infection Fatality Rate
Re	Effective reproduction number
PAHO	Pan-American Health Organization
SNV	Single-nucleotide variant
RBD	Receptor-binding domain
NTD	N-terminal domain
NSP13	Nonstructural protein 13
NSP12b	Nonstructural protein 12 b
ORF3a	Open reading frame 3a
NSP3	Nonstructural protein 3
NSP2	Nonstructural protein
S	Spike
